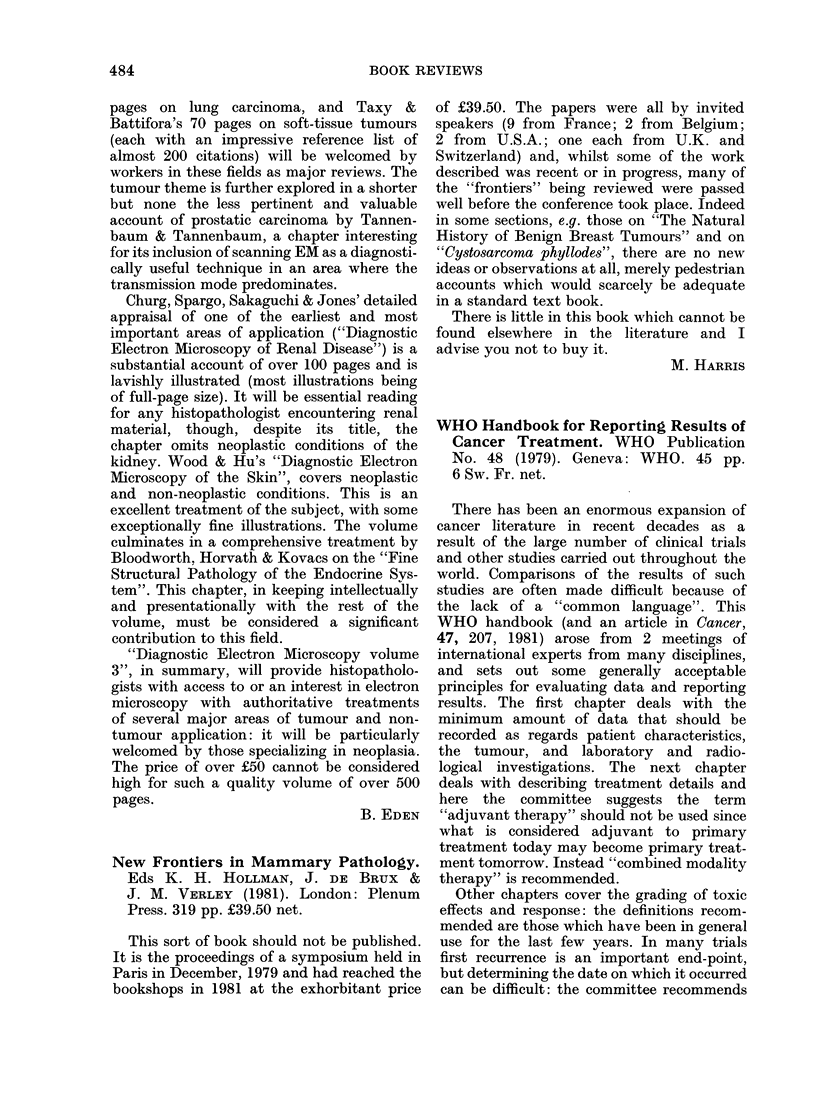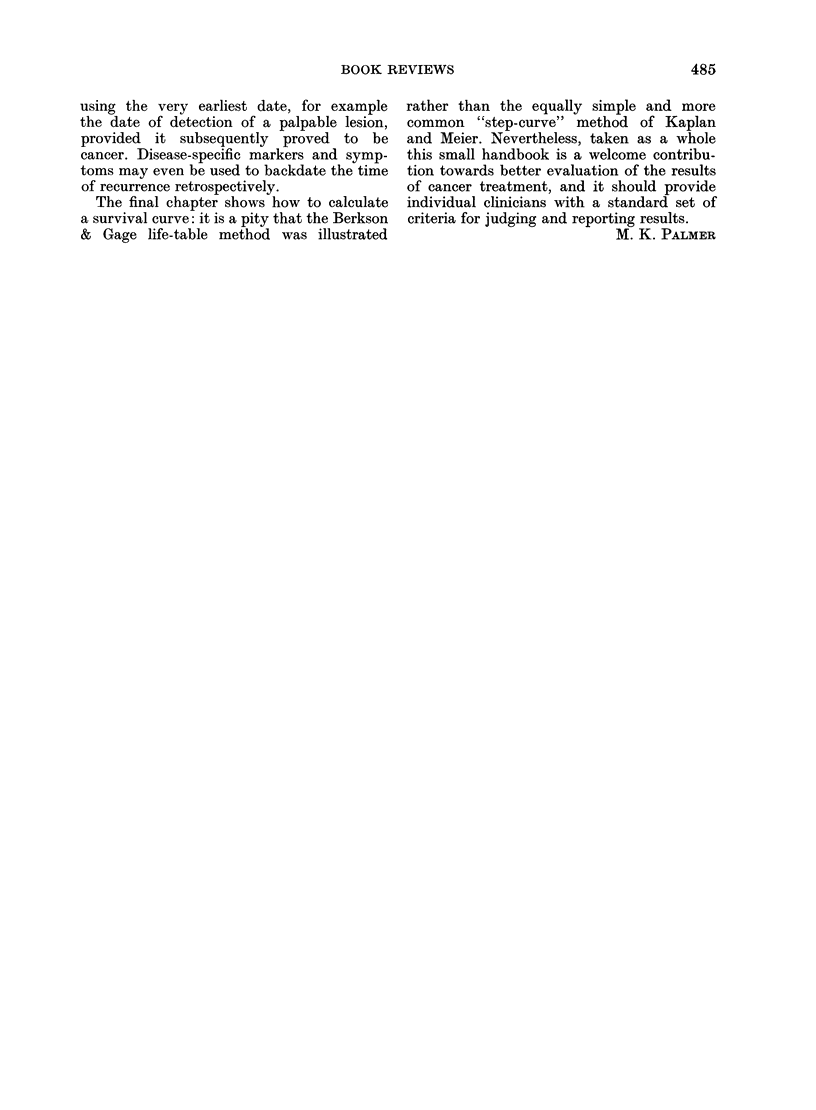# WHO Handbook for Reporting Results of Cancer Treatment

**Published:** 1982-03

**Authors:** M. K. Palmer


**WHO Handbook for Reporting Results of Cancer Treatment.** WHO Publication No. 48 (1979). Geneva: WHO. 45 pp. 6 Sw. Fr. net.

There has been an enormous expansion of cancer literature in recent decades as a result of the large number of clinical trials and other studies carried out throughout the world. Comparisons of the results of such studies are often made difficult because of the lack of a "common language". This WHO handbook (and an article in *Cancer*, **47,** 207, 1981) arose from 2 meetings of international experts from many disciplines, and sets out some generally acceptable principles for evaluating data and reporting results. The first chapter deals with the minimum amount of data that should be recorded as regards patient characteristics, the tumour, and laboratory and radiological investigations. The next chapter deals with describing treatment details and here the committee suggests the term "adjuvant therapy" should not be used since what is considered adjuvant to primary treatment today may become primary treatment tomorrow. Instead "combined modality therapy" is recommended.

Other chapters cover the grading of toxic effects and response: the definitions recommended are those which have been in general use for the last few years. In many trials first recurrence is an important end-point, but determining the date on which it occurred can be difficult: the committee recommends using the very earliest date, for example the date of detection of a palpable lesion, provided it subsequently proved to be cancer. Disease-specific markers and symptoms may even be used to backdate the time of recurrence retrospectively.

The final chapter shows how to calculate a survival curve: it is a pity that the Berkson & Gage life-table method was illustrated rather than the equally simple and more common "step-curve" method of Kaplan and Meier. Nevertheless, taken as a whole this small handbook is a welcome contribution towards better evaluation of the results of cancer treatment, and it should provide individual clinicians with a standard set of criteria for judging and reporting results.

M. K. Palmer